# Degradation of Poly(ε-caprolactone) by thermophilic *Streptomyces thermoviolaceus subsp. thermoviolaceus* 76T-2

**DOI:** 10.1186/2191-0855-3-8

**Published:** 2013-01-29

**Authors:** Te-Kuan Chua, Min Tseng, Mei-Kwei Yang

**Affiliations:** 1 Department of Life Science, Fu Jen University, Hsin Chuang, Taipei, Taiwan; 2 Bioresource Collection and Research Center, Food Industry Research and Development Institute, Hsinchu, Taiwan

**Keywords:** Poly(ε-caprolactone) (PCL), *Streptomyces thermoviolaceus subsp. thermoviolaceus*, PCL depolymerases, Chi25 chitinase

## Abstract

A thermophilic *Streptomyces thermoviolaceus subsp. thermoviolaceus* isolate 76T-2 that can degrade poly(ε-caprolactone) (PCL) was isolated from soil in Taiwan. Isolate 76T-2 grew well in urea fructose oatmeal medium and exhibited clear zones on agar plates containing PCL, indicating the presence of extracellular PCL depolymerases. The PCL powder present in culture medium was completely degraded within 6 h of culture at 45°C. Two PCL-degrading enzymes were purified to homogeneity from the culture supernatant. The molecular weights of these two enzymes were estimated to be 25 kDa and 55 kDa, respectively. A portion of the N-terminal region of the 25-kDa protein was determined, and the sequence Ala-Asn-Phe-Val-Val-Ser-Glu-Ala thus obtained was identical to that of A_64_-A_71_ of the Chi25 chitinase of *Streptomyces thermoviolaceus* OPC-520. The 25-kDa protein was shown to also degrade chitin, suggesting that isolate 76T-2 has the ability to degrade both PCL and chitin.

## Introduction

Polycaprolactone (PCL), a biodegradable synthetic aliphatic polyester, is a polymer of ε-caprolactone (Tokiwa et al. 1976, Tokiwa and Suzuki 1977, Benedict et al. 1983a). Due to its favorable physical properties such as low melting point (60°C), low glass transition temperature (−60°C), hydrophobicity, and crystallinity which resemble those of conventional plastics, PCL is being used as a biodegradable substitute for non-degradable plastics (Tokiwa and Calabia 2004, Tokiwa et al. 2009). Since PCL is thermostable and biocompatible for tissue engineering, drug release, and wound dressing, it has a great potential in industrial and medical applications (Sinha et al*.*^2004^, Lenz and Marchessault 2005, Sun et al*.*^2006^, Shah et al. 2008).

Degradation of PCL may take place in soil, water, and compost by microorganisms (Tokiwa et al. 1976, Nishida and Tokiwa 1993, Klingbeil et al. 1996, Abou-Zeid et al. 2001, Ponsart et al. 2001, Federle et al. 2002, Abou-Zeid et al. 2004, Nakasaki et al. 2006, Tokiwa et al. 2009). Most PCL-degrading fungi are found in genera *Penicillium* and *Aspergillus* (Tokiwa et al. 1976, Benedict et al. 1983b, Sanchez et al. 2000)*,* and most PCL-degrading bacteria belong to the genus *Clostridium* (Abou-Zeid et al. 2001, 2004). A thermophilic *Streptomyces* isolated from compost was found to have a higher PCL-degrading activity than thermotolerant *Aspergillus* (Nakasaki et al. 2006). Since composting at high temperature is an ideal method for recycling biodegradable plastics by thermophilic microorganisms (Tokiwa et al. 2009), isolation of thermal resistant PCL-degraders is desired.

Among the various thermophilic microorganisms, some actinomycetes have been shown to produce enzymes that hydrolyze polyesters at high temperature (Klingbeil et al. 1996, Calabia and Tokiwa 2004, Tokiwa and Calabia 2004). We have isolated many polyester-degrading thermophilic actinomycetes from different environments in Taiwan (Tseng et al. 2007). Some of them can degrade either polyhydroxybutyrate (PHB), PCL, or polyethersulfone (PES); others can degrade two or all three of these polyesters. Among the 198 PCL-degrading actinomycetes we have isolated, *Streptomyces thermoviolaceus subsp. thermoviolaceus* 76T-2, isolated from soil, has the highest growth and PCL hydrolysis rates. To be able to use it in an industrial scale, we characterized the organism and purified the PCL-degrading enzymes from this organism.

## Materials and methods

### Screening of PCL-degrading thermophilic actinomycetes

Thermophilic actinomycetes were isolated from soil samples as described previously (Tseng et al. 2007). The samples were plated on modified HV agar plates (humic acid, 1.0 g; yeast extract, 3.0 g; KCl, 1.7 g; FeSO_4_·7H_2_O, 0.01 g; Na_2_HPO_4_, 0.5 g; MgSO_4_·7H_2_O, 0.05 g; CaCO_3,_ 0.02 g; cycloheximide, 50 mg; nalidixic acid, 20 mg; 0.5 mg each of thiamine-HCl, riboflavin, niacin, pyridoxine-HCl, inositol, Ca-pantothenate, p-aminobenzoic acid, and 0.25 mg of biotin; agar, 18.0 g; distilled water 1 L, pH 7.2), and the plates were incubated at 45°C for 7–10 days. The powdery colonies with branched hyphae on the plates were then picked and streaked on International Streptomyces Project (ISP) 3 agar plates (oatmeal, 20 g; 1 mg each of FeSO_4_·7H_2_O, MnCl_2_·H_2_O and ZnSO_4_·7H_2_O; agar, 18 g in 1 L of distilled water, pH 7.2) and incubated at 45°C for 3–7 days for identification. The PCL-degradation ability of the isolates was detected by the presence of a clear zone around the colonies on agar plates containing emulsified PCL. PCL agar plates were made by dissolving one gram of PCL powder in 50 ml of methylene chloride and then emulsified into 50 ml basal medium containing (per liter) yeast extract, 0.1 g; FeSO_4_·7H_2_O, 10 mg; MgSO_4_·7H_2_O, 0.2 g; (NH_4_)_2_SO_4,_ 1 g; CaCl_2_·2H_2_O, 20 mg; NaCl, 0.1 g; Na_2_MoO_4_·2H_2_O, 0.5 mg; NaWO_4_·2H_2_O, 0.5 mg; MnSO_4_·H_2_O, 0.6 mg; and dishwashing liquid, 1 ml (Poas, Nice Co., Taiwan). Methylene chloride was then evaporated in a hume hood.

### Identification of PCL-degrading thermophilic actinomycetes

To identify isolate 76T-2, scanning electron microscopy was first performed. After growth on oatmeal agar (ISP3 medium) plates at 45°C for 7 days, a representative colony was isolated, fixed in 4% osmium tetroxide, dehydrated through a graded ethanol series, dried to critical point, coated with gold, and then examined with a scanning electron microscope (Itoh et al. 1989). Morphological characteristics were assessed by using 7-day cultures grown at 45°C on yeast extract-malt extract agar (ISP 2 medium), oatmeal agar (ISP 3 medium), inorganic salts-starch agar (ISP 4 medium), and glycerol-asparagine agar (ISP 5 medium) (Shirling and Gottlieb 1966). The ISCC-NBS Color-Name Charts were used for color designations of substrate mycelium and aerial mass. 76T-2 cells for chemotaxonomic and molecular studies were harvested after incubation in yeast-glucose broth (yeast extract 1.0%, glucose 1.0%, w/v) for 7 days at 45°C. Isomers of diaminopimelic acid (A2pm) and sugars in whole-cell lysates and cell walls were determined as described previously (Hasegawa et al. 1983).

### Phylogenetic analysis

To determine the nucleotide sequence of the 16S rRNA gene, genomic DNA was extracted from 5-day cultured cells using the QIAGEN® Genomic DNA Kit. A portion of the 16S rRNA gene was amplified by PCR as described by Nakajima et al. (Nakajima et al. 1999) and then sequenced directly on an ABI automatic DNA sequencer (Model 3730) using the BigDye Terminator V3.1 kit (Applied Biosystems). The resulting 16S rDNA sequence was compared to the sequences obtained from the EzTaxon server (Chun et al. 2007) to identify 76T-2.

### Growth and PCL-degrading ability of *S. thermoviolaceus* subsp. *thermoviolaceus* 76T-2

The growth characteristic was determined by growing 76T-2 cells in Luria Bertani (LB), UF (15 g of fructose, 10 g of peptone, 10 g of yeast extract, 2 g of urea, 20 mg of CaCl_2_·2H_2_O_,_ 200 mg of MgSO_4_·7H_2_O in one liter of distilled water), and Urea Fructose Oatmeal (UFO) media. The optimal growth temperature was assessed using UF medium. For examination of PCL-degrading enzymes, 76T-2 cells were grown at 45°C in UF medium to stationary phase and then transferred to basal medium containing 0.1% (w/v) PCL for various lengths of time. To assay extracellular PCL-degrading enzymes, aliquots of culture supernatant were assessed for the decrease in turbidity of the emulsified PCL. To quantify PCL-degrading activity, 1 ml of an overnight liquid culture in UF medium was mixed with 20 ml of basal medium containing 0.3% PCL and incubated at 30-50°C for 14 h. The PCL-degrading activity was measured spectrophotometrically at 650 nm.

### Purification of PCL depolymerase

For isolation of extracellular PCL depolymerases, 76T-2 cells were grown in basal medium containing PCL as carbon source at 45°C for 12 h with shaking. The cells were removed by centrifugation at 12,000 x g for 20 min at 4°C, and the supernatant was used for enzyme preparation. Crude enzymes were precipitated by adding solid ammonium sulfate with continuous stirring at 4°C for 1 h and then pelleted by centrifugation. The precipitate was dissolved in 50 mM Tris–HCl buffer (pH 7.2) and dialyzed against four volumes of the buffer. Protein concentration was determined using the Lowry method with bovine serum albumin as a standard. Isolated enzymes were subjected to 10% sodium dodecyl sulfate-polyacrylamide gel electrophoresis (SDS-PAGE). PCL-degrading activity was detected by zymography by separating the proteins in a native polyacrylamide gel. After electrophoresis, the gel was placed on another polyacrylamide gel containing 0.1% PCL or chitin and incubated at 45°C for 30 min. Active protein bands were detected as clear zones. The gel was then stained with Coomassie brilliant blue R-250 to determine the sizes of the proteins with PCL-degrading activity.

## Results

### Isolation and identification of PCL-degrading *S. thermoviolaceus* subsp. *thermoviolaceus* isolate 76T-2

One gram of each soil sample was suspended in 9 mL of sterilized distilled water. A serially diluted 0.1-mL aliquot of each sample was plated on a modified HV agar plate and incubated at 45-50°C for 7–10 days. Many powdery and irregular colonies with branched hyphae were seen in most cultures. Some of these isolates were found to degrade PCL when they were subcultured on oatmeal agar plates containing PCL, and an isolate designated 76T-2 was found to have the highest PCL-degrading activity .

**Figure 1:**
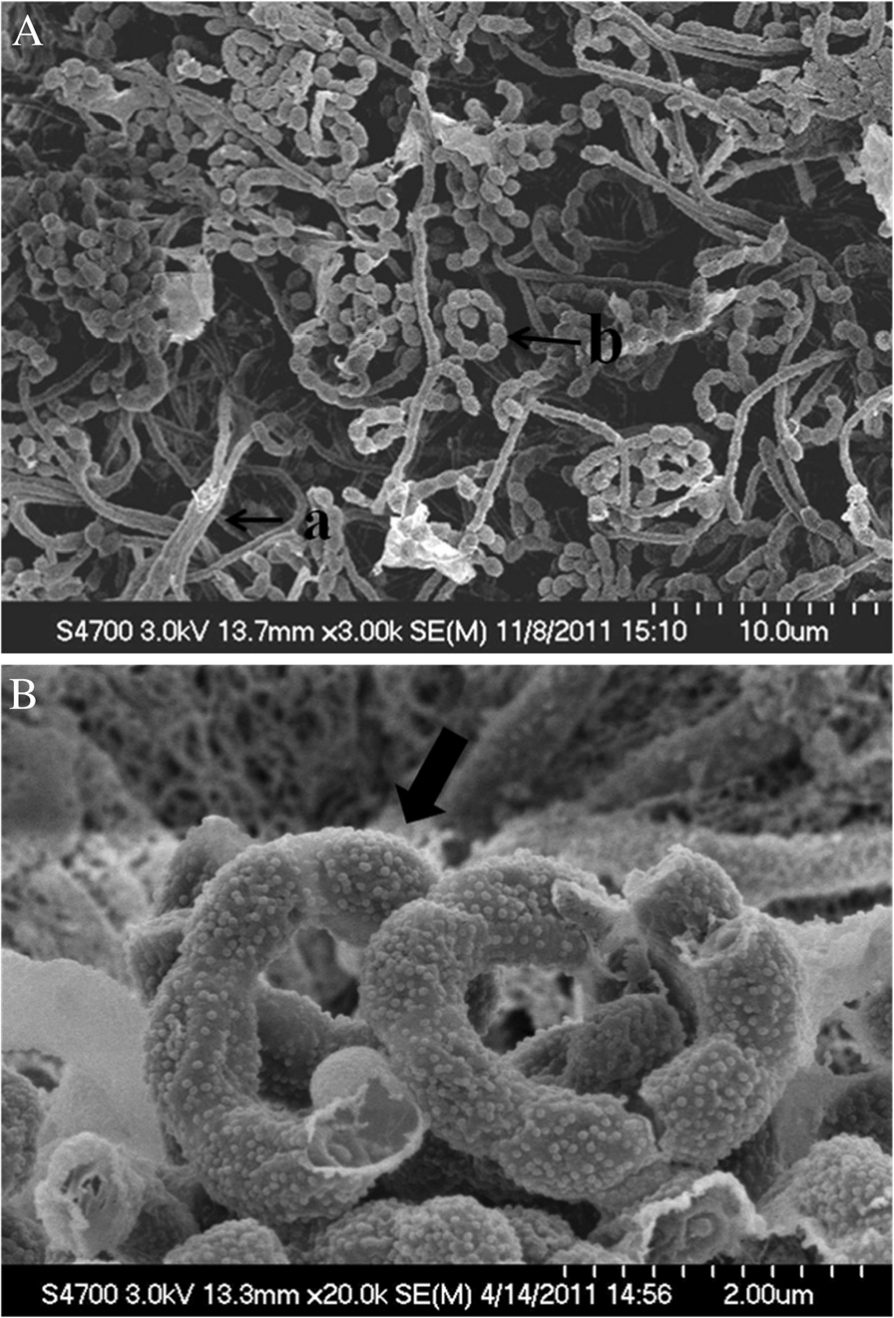
**Scanning electron micrographs of *Streptomyces thermoviolaceus subsp. thermoviolaceus *isolate 76T-2.** Aerial mycelia with chains of spores in the form of terminal, closed or compact spirals were observed after 7 days of growth on oatmeal agar plates at 45°C.

Isolate 76T-2 produced round, pale white cottony colonies on oatmeal agar plates. Analyses of its peptidoglycan cell wall revealed that the major constituent was LL-diaminopimelic acid (LL-DAP). No sugars were detected. The cell wall was thus determined to be chemotype I (Lechevalier and Lechevalier 1970). Its aerial mass on ISP medium 4 was grayish-brown or grayish-purple, and substrate mycelia were brownish-black, light brown, or purplish-white. On ISP medium 2, isolate 76T-2 showed robust growth; rates of growth on ISP media 3, 4, and 5 were moderate. Sporulation of isolate 76T-2 on ISP medium 2 was good, moderate on ISP 3 and 5 media, but poor on ISP medium 4. Mature spore chains were spiral-shaped with a warty surface (Figure 1). Based on these observations, isolate 76T-2 was considered a *Streptomyces* (Lechevalier and Lechevalier 1970). The 16S rRNA sequence of 76T-2 (GenBank accession no. KC470043) was aligned with those of *Streptomyces* and found to be 99.9% homologous to that of *Streptomyces thermoviolaceus* subsp. *thermoviolaceus.* The isolate *Streptomyces thermoviolaceus* subsp. *thermoviolaceus* 76T-2 (BCRC16931) has been deposited in the Bioresource Collection and Research Center, Food Industry Research and Development Institute, Hsinchu, Taiwan.

### Growth and PCL-degrading activity of *S. thermoviolaceus* subsp. *thermoviolaceus* isolate 76T-2

**Figure 2:**
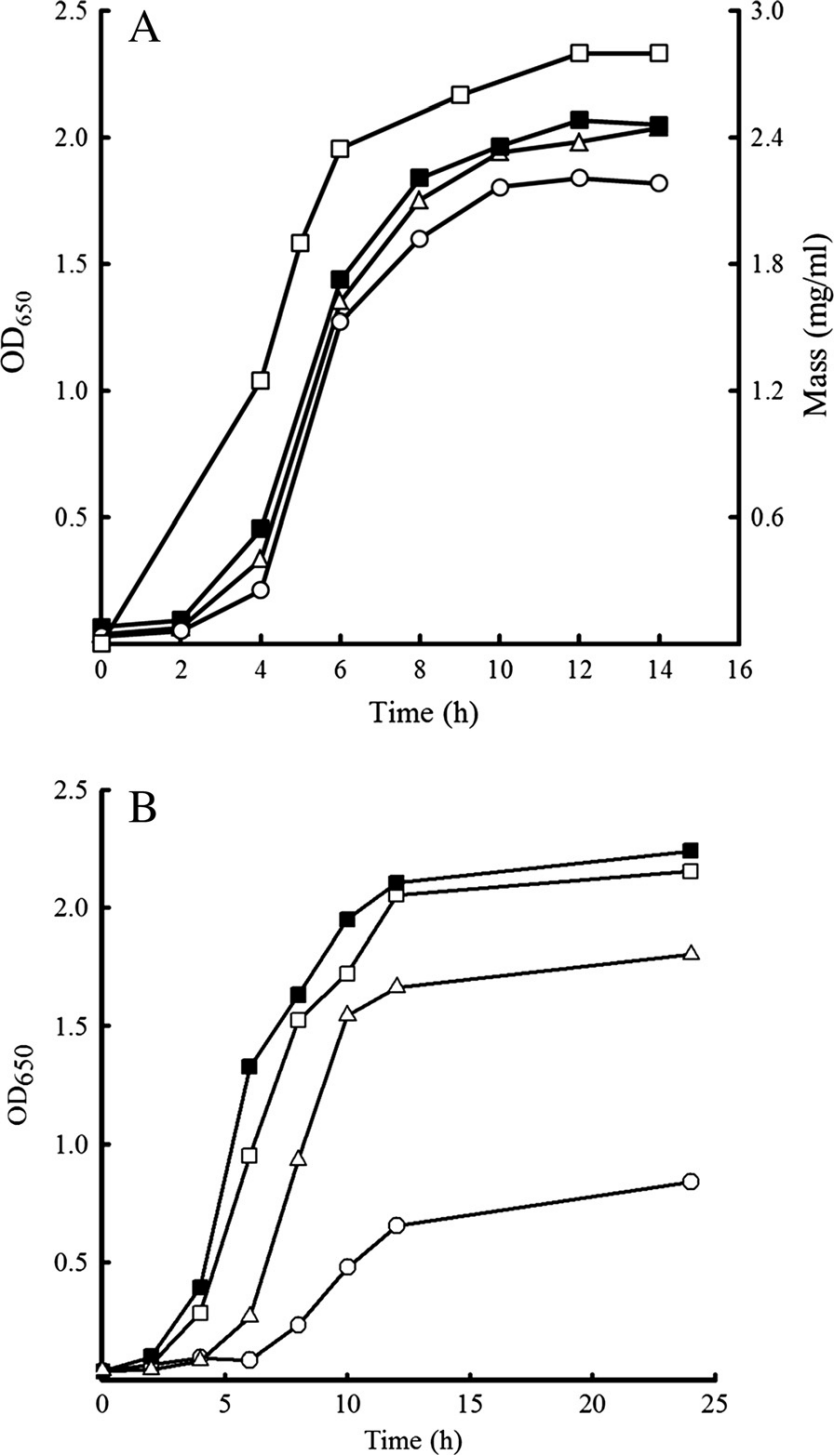
**Growth of *Streptomyces thermoviolaceus subsp. thermoviolaceus *isolate 76T-2 in various media and different temperatures.** (**A**) Growth curve of 76T-2 in UFO (−■ -), UF(─△ ─), and LB (─○ ─) media at 45°C. The cell mass (─ □ ─) and OD_650_ values were measured at different time points as indicated. (**B**) Growth curve of 76T-2 at 45°C (−■ -), 40°C (─ □ ─), 50°C (─ △ ─), and 30°C (─ ○ ─) in UF medium.

76T-2 cells were cultured in UFO, UF, and LB media to determine their growth requirements. The cell density (absorbance at 650 nm) and dried cell mass of the cultures were measured every two hours for 14 hours (Figure 2A). A rapid growth was observed after 2 h, and the growth peaked after 12 h of incubation in all three media. Maximum growth was observed (OD_650_ = 2.0, cell mass = 2.8 mg/ml) after 12 h of incubation in UF medium at 40°C or 45°C. A slower growth rate was seen after 12 h (OD_650_ = 1.6) and 24 h (OD_650_ = 1.7) of incubation at 50°C. 76T-2 cells did not grow well at 30°C. These results (Figure 2B) suggest that the optimal growth temperature for 76T-2 is 45°C.

**Figure 3:**
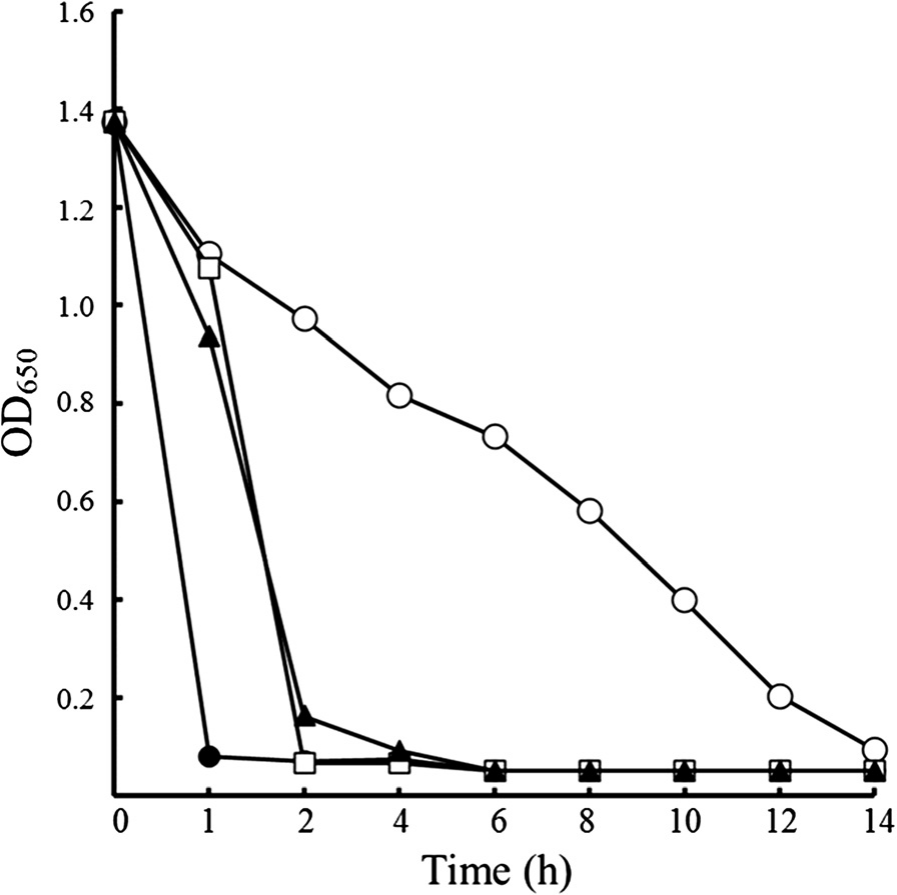
**Effect of temperature on PCL degradation by *Streptomyces thermoviolaceus subsp. thermoviolaceus *isolate 76T-2.** 76T-2 cells were cultured at 30°C (─ ○ ─), 40°C (−● -), 45°C (─ ▲ ─), and 50°C (─ □ ─) in basal medium containing 0.3% emulsified PCL powder as the major carbon source. At 2 h intervals, after centrifugation at 12,000Xg for 30 min, the PCL depolymerase activity was assayed by determining the O.D_650_ value of the culture supernatant.

The effect of temperature on PCL degradation by 76T-2 was then investigated. 76T-2 cells were grown in UF medium for 12 h at 45°C and then in basal medium containing 0.3% PCL at a temperature between 30°C and 50°C. The PCL hydrolytic activity was assayed every 2 h for 14 h by measuring the turbidity of the culture. Changes in PCL turbidity as indicated by OD_650_ values due to PCL degradation at various temperatures are shown in Figure 3. The hydrolysis of PCL was noticeable at the begining (OD_650_ = 1.4) of culture and was maximal (OD_650_ = 0.05) at 1 h of incubation when 76T-2 cells were grown at 40°C. No further increase in PCL degradation was observed afterward.

At 45°C, a lower degree of PCL hydrolysis (OD_650_ = 0.95) was observed after 1 h of culture, but a significant degradation (OD_650_ = 0.15) of PCL was seen at the 2 h time point. The PCL degradation reached the maximum (OD_650_ = 0.05) after 4 h of incubation. A similar trend of PCL degradation was observed at 50°C with an OD_650_ = 1.1 at 1 h and OD_650_ = 0.05 at 2 h and no further increses in PCL degradation afterward. However, when the temperature was changed to 30°C, a slow (OD_650_ = 1.1 after 1 h incubation) and gradual increase in PCL degradation rate with an OD_650_ = 0.1 at 14 h of incubation was observed. These results suggest that the optimal temperature for PCL degradation was 40°C.

### Isolation of PCL depolymerases

**Figure 4:**
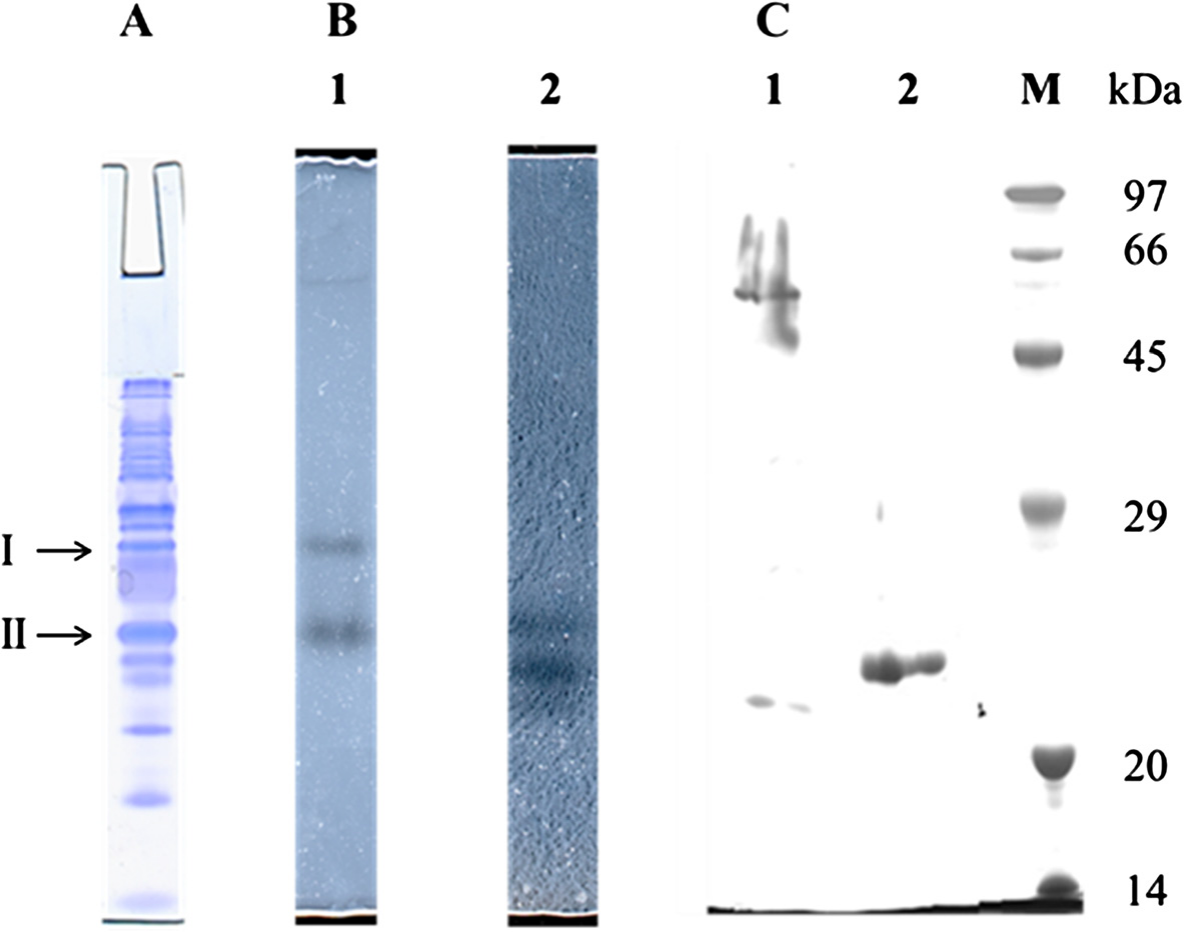
**PAGE and zymography of ammonium sulfate precipitate of 76T-2 culture supernatant.** Electrophoresis was carried out in a native 8% polyacrylamide gel (**A**). Zymography was performed by overlaying the gel on another native 8% polyacrylamide gel containing PCL (**B**1) or chitin (**B**2). The proteins in bands with enzymatic activity were isolated and electrophoresed in a 10% SDS-polyacrylamide gel which was then stained with Coomassie brilliant blue R-250 (**C**): lane 1, purified 55-kda PCL depolymerase I; lane 2, purified 25-kda PCL depolymerase II.

The PCL degradation enzymes were then isolated from culture supernatant of 76T-2 cells grown under optimal conditions. Solid ammonium sulfate was added to the supernatant. At 60% (w/v) concentration, maximum protein precipitation was achieved. When the precipitate was analyzed by electrophoresis in an 8% native polyacrylamide gel, multiple protein bands were found (Figure 4A). Two bands of clear zone due to PCL hydrolysis were seen after performing a zymography by overlaying the gel on another 8% polyacrylamide gel containing PCL (Figure 4B, lane 1). The protein I and protein II with PCL depolymerase activity were eluted and electrophoresed on another 10% SDS polyacrylamide gel. Single bands with a molecular mass of approximately 55 kDa (protein I) and 25 kDa (protein II), respectively were observed (Figure 4C, lanes 1 and 2). The sequence of the first eight N-terminal amino acid residues of the purified 25-kDa PCL depolymerase was determined and found to be Ala-Asn-Phe-Val-Val-Ser-Glu-Ala. A similarity search of the DDJB sequence database indicated that this sequence was identical to amino acids A_64_-A_71_ of the Chi25 chitinase of *Streptomyces thermoviolaceus* OPC-520.

To investigate chitin degradation activity of the PCL depolymerases, the 60% ammonium sulfate precipitate was analyzed in an 8% native polyacrylamide gel and then overlaid on another gel containing chitin. A clear zone due to chitin hydrolysis was seen at the place corresponding to protein II (25-kDa protein) but not protein I (55-kDa protein) (Figure 4B, lane 2). This result indicated that the 25-kDa PCL depolymerase secreted by 76T-2 also had the ability to degrade chitin, wheras the 55-kDa protein was not capable of degrading chitin.

## Discussion

Polycaprolactone (PCL), an important polymer due to its strong mechanical properties, biodegradability, and miscibility with a number of other polymers (Kim and Rhee 2003, Labet and Thielemans 2009), has been investigated for its degradation in terrestrial and aquatic environments. A number of PCL-degrading bacterial and fungal strains of *Alcaligenes, Clostridium, Aspergillus, Penicillium*, *Fusarium*, and *Streptomyces* have been isolated (Tokiwa et al. 1976, Benedict et al. 1983a, 1983b, Tokiwa et al. 2009). In this study, we isolated a PCL-degrading bacterium from soil and identified it as *Streptomyces thermoviolaceus subsp. thermoviolaceus* based on its morphology, growth characteristics, and 16S rRNA gene sequence. This isolate was designated 76T-2. It grew well and formed clear zones on PCL emulsified agar plate at 45-50°C in one day, indicating that it is a fast-growing thermophile. Its PCL degradation ability was confirmed by demonstrating that the culture supernatant contained PCL-degrading enzymes.

Two PCL depolymerases of approximately 55 and 25 kDa in size were detected. Although several extracellular PCL depolymerases have been isolaed (Murphy et al. 1996, Oda et al. 1997, Li et al. 2012), few of them have been fully characterized*.* Therefore, there is no previous report on the size range of bacterial or fungal PCL depolymerases. To our knowledge, this is the first report describing the characterization of a purified PCL depolymerase from a thermophilic *Streptomyces*. Based on its N-terminal amino acid sequence and activity against chitin, we consider the 25 kDa enzyme a chitinase with PCL-degrading activity.

PCL can be degraded by two types of esterases: One is lipase, and the other is cutinase. One example of lipase with PCL-degrading activity is the lipase from *Alcaligenes faecalis* (Oda et al. 1997). Cutinase is a serine hydrolase which breaks ester bonds in cutin. *Fusarium* is one example of bacteria producing such enzyme (Murphy et al. 1996). Chitin is a polymer of β-1,4-N-acetylglucosamine in insects, crustaceans, fungi, and algae. The finding that the 25-kDa PCL depolymerase from 76T-2 had chitinase activity is novel as no other PCL depolymerases have been found to have such activity. Therefore, the 25-kDa enzyme isolated from 76T-2 may be a novel type of PCL depolymerase. Since isolate 76T-2 does not form clear zones on PHB-containing agar plate and there is no report that PCL depolymerases have PHA degrading activity, it is likely that the PCL depolymerases of 76T-2 do not degrade PHA.

Zymographic studies revealed another PCL depolymerase with a molecular mass of 55 kDa, and the purified 55-kDa protein was able to degrade PCL. Although numerous attemps were made, we were unable to determine the N-terminal amino acid sequence of this protein. Since the molecular mass of the *Fusarium* PCL depolymerase is 25 kDa and that of lipases are larger (30 kDa) (Murphy et al. 1996), we focused our study on the 25-kDa PCL depolymerase.

Four chitinases (Chi40, Chi35, Chi30 and Chi25) have been identified in *Streptomyces thermoviolaceus* OPC-520 (Tsujibo et al. 1993). Based on amino acid sequence similarity of their catalytic domains, Chi40 and Chi30 are considered as members of family 18, and Chi35 and Chi25 are classified as family 19 of glycosyl hydrolases (Tsujibo et al. 2000b). Chi40 is the major chitinase of *Streptomyces thermoviolaceus* OPC-520 secreted into the culture medium. Since Chi25 and Chi35 are produced in very low levels, they are postulated to function as antifungal agents rather than as major enzymes to digest chitin (Tsujibo et al. 2000a). Genes encode for Chi25 and Chi35 have been cloned and found to be arranged in tandem. The deduced amino acid sequences of the catalytic domains of Chi25 and Chi35 are highly similar to each other. In addition to the catalytic domain, Chi35 has a N-terminal domain for polysaccharide binding (Tsujibo et al. 2000b) and sequences similar to certain domains of bacterial polymer-degrading enzymes such as xylanase I of *S*. *thermoviolaceus* OPC-520 (Tsujibo et al. 1997).

Although chitinases from many *Streptomyces* species have been extensively studied with respect to their structure, function, and regulation of gene expression (Saito et al. 1999, Kim et al. 2003, Saito et al. 2003, Okazaki et al. 2004, Yano et al. 2008, Hoang et al. 2011), few studies have been conducted on the degradation of biodegradable plastic by chitinases. In this study, we found that the 25-kDa PCL-degrading enzyme is homologous to the chitinase (Chi25) of *Streptomyces thermovio*laceus OPC-520 (GenBank accession number AB016843) (Tsujibo et al. 2000a, 2000b). Further study of the 25-kDa PCL-degrading enzyme from 76T-2 will advance our knowledge about the PCL depolymerases of *S. thermoviolaceus.*

## Competing interests

The authors declare that they have no competing interests.
